# The temporal variation of CH_4_ emissions embodied in Chinese supply chains, 2000–2020

**DOI:** 10.1038/s41598-024-62979-z

**Published:** 2024-05-29

**Authors:** Jiaxi Wu, Mengxin Chen, Xialing Sun, Zheng Meng

**Affiliations:** 1 School of Management, China University of Mining & Technology, Beijing, 100083 People’s Republic of China; 2 China Energy Engineering Group Anhui Electric Power Design Institute Co., Ltd, Beijing, 230601 People’s Republic of China; 3 School of Public Health, Shandong Second Medical University, Weifang, 261053 People’s Republic of China; 4 School of Economics and Management, Shihezi University, Shihezi, Xinjiang 832000 People’s Republic of China

**Keywords:** Methane (CH_4_) emissions, Input–output analysis, Structure path analysis, Supply chains, China, Ecology, Environmental sciences, Environmental social sciences

## Abstract

Although the issue of embodied pollutants in China’s supply chains has garnered increasing attention, the dynamic changes occurring within them are unclear. Several existing studies analyze one-year or short-term data in supply chain. China’s overall CH_4_ emissions have risen from 41.1 Tg in 2000 to 60 Tg in 2020, so conducting long-term analyses can yield a deeper understanding of the dynamic changes across the entire supply chain from production to consumption. This study uses the environmentally extended input–output analysis (EEIOA) and structural path analysis (SPA) methods to investigate the dynamic variation of China’s embodied CH_4_ emissions in 20 industry sectors from 2000 to 2020, aiming to determine the key supply chain and key sectors. The results reveal that from the final demand perspective, consumption, investment and export drove 52.1%, 32%, and 15.9% of embodied CH_4_ emissions in 2020. The sector with the highest embodied CH_4_ emissions has changed from “Agriculture” in 2000 to “Construction” in 2010 to “Other service and activities” in 2020. The top listed supply chain path of embodied CH_4_ emissions has also evolved (starting from production to consumption) from “Agriculture → Rural consumption” in 2000 to “Agriculture → Food and tobacco → Urban consumption” in 2010 to “Agriculture → Urban consumption” in 2020. Notably, the high-ranked path, “Agriculture → Food and tobacco → Rural consumption”, shows that the embodied CH_4_ emission flowing between agriculture and the food industry cannot be ignored. The supply chain path “Coal Mining → Nonmetal Mineral Products → Construction → Capital Formation” has risen from 17th in 2000 to 3rd in 2020. Thus, it is necessary to control CH_4_ emissions from sectors upstream, which are predominantly influenced by the construction industry, and a coordinated effort between sectors is also required to effectively reduce emissions. By 2020, the CH_4_ emissions driven by urban consumption were 3.1 times that of rural consumption. This study provides a comprehensive analysis of China's supply chain over the past two decades. In particular, it suggests policy interventions by controlling critical supply chain paths and key sectors associated with embodied CH_4_ emission, thereby facilitating the coordinated reduction of anthropogenic CH_4_ emissions.

## Introduction

The short-lived greenhouse gas (GHG) methane (CH_4_) ranks second only to carbon dioxide (CO_2_) in terms of global emissions, but it is more active in the atmosphere and with higher heat absorbing capacity than CO_2_^1^. By keeping global temperature decline by 1.5 °C, the IPCC’s special report emphasized strengthening global mitigation of non-CO_2_ GHG, especially CH_4_^2^. However, CH_4_ emissions have more than doubled since the pre-industrial level (1877 ± 2 ppb in 2019), and they continue to rise ^3,4^. The world needs to reduce CH_4_ emissions by at least 30% from 2020 levels by the year 2030 to halt global temperatures rising by 1.5 °C^2,5^. Since CH_4_ has a short lifetime (around 12 years), reduction activities can affect its concentration in the atmosphere relatively quickly. While CO_2_ remains in the atmosphere for a long time (50–200 years), reducing it requires longer periods of time to be effective^6^. Therefore, prioritizing CH_4_ reduction is considered a direct and effective means of slowing down global warming in the short term. As part of the Global Methane Pledge, over 100 nations have committed to reducing global CH_4_ emissions by at least 30% from 2020 levels by 2030, further highlighting the increasing attention given to CH_4_ emissions in international society^7,8^.

CH_4_ plays an important role in China's overall composition of GHG emissions^9^. According to China’s Initial and Third National Communication on Climate Change^10,11^, CH_4_ emissions in 1994, 2005, and 2014 were 34.3 Tg, 44.5 Tg, and 55.3 Tg, representing 19.4%, 13.2%, and 10.4% with global warming potential (GWP) in 21. CH_4_ emissions in China were equivalent to 1.2 billion tons of CO_2_ (including land use change and forestry) in 2014, exceeding those in many developed countries^12^. In light of China’s prominence in CH_4_ emissions, only considering CO_2_ cannot fully reflect China’s overall pattern of GHG emissions. Consequently, reducing CH_4_ emissions is a prerequisite for achieving comprehensive GHG reductions and demonstrating active compliance with international commitments.

Previous studies have extensively explored CH_4_ emission on national^12^, regional^13^ and sectoral^14^ scales from the perspective of production. However, production-based inventory does not render a complete visualisation of the entire supply chain. Thus, in recent years, CH_4_ emissions in resources and emissions have been further explored from the perspective of consumption (or implementation or footprint)^15^. A large number of studies attributed the resources and emissions from production to consumption^16^. However, it still remains unclear how consumption activities induce CH_4_ emissions via intermediate steps in the supply chain. As a supplement, environmental extended input–output analysis (EEIOA) is considered the most widely accepted macroeconomic method to analyse the connection between industry sectors and environmental flows in terms of environmental emissions^17,18^. The concept, originally proposed by Leontief, is a powerful tool to establish the relationship between final demand and environmental emissions. This method has been increasingly adopted by researchers to examine the relationship between economic activities and the ecological environment from a consumption perspective at the global scale^19,20^, national scale^21,22^, inter-provincial scale^23,24^ and even at the city scale^25^.

In analysing the impact of driving factors on a certain indicator, the Index Decomposition Analysis (IDA) method with low data requirements is often used to analyse the direct impact of driving factors on a certain indicator. However, this method cannot show the relationship with of different sectors^26^. While the Structural Decomposition Analysis (SDA) method can comprehensively analyse the direct or indirect factors that driving factors have on a certain indicator^23^. After reviewing existing research findings, it was found that most of the related studies have focused on discussing the driving factors of CH_4_ emissions, without further clarifying the transmission pathways of embodied CH_4_ emissions among various industrial sectors. Structural path analysis (SPA) enables the tracing of flows throughout the entire supply chain, facilitating an understanding of the role played by final demand in driving CH_4_ emissions and aiding in the identification of critical transmission sectors and supply chain paths. Research has demonstrated the utility of the SPA method for illustrating the embodied resources and environmental factors in supply chains^27,28^. Notably, China’s national economic system features an extremely complex industrial chain, and SPA has been increasingly adopted to explore the flow of natural resources and GHGs from the production sector through the intermediate consumption to the final demand. Moreover, Meng et al.^29^ combined the environmental input–output model with SPA to mitigate primary PM2.5 emissions in China from both production and consumption perspectives and their linkages. Wang et al.^30^ analysed the flows of natural resources within China’s economic system from raw material extraction to final product production. They revealed that the most critical supply chain paths originate in the resource extraction sector and eventually end in the construction sector^30^. Furthermore, Zhang et al.^15^ estimated the non-CO_2_ GHG emissions for China in 2012 from the consumption perspective. The research has also focused on critical embodied emission paths and key industry sectors in supply chains for mitigating non-CO_2_ GHG emissions in China’s economic systems using the SPA method^15,31^. These studies provide a reference for the national non-CO_2_ GHG emissions reduction strategy. Although previous studies have linked the non-CO_2_ GHG emission of the production sector to final demand users^32,33^, primarily analyse one-year or short-term time series data to understand CH_4_ emissions. However, there is still a lag in our knowledge of the temporal changes experienced by embodied CH_4_ emissions in Chinese supply chains. With rapid economic growth and carbon neutrality goals raised in China in recent years, the critical supply chains that contribute most strongly to embodied CH_4_ emissions may shift dramatically. Therefore, it is critical to identify the dynamic variations in supply chain paths that drive embodied emissions. Managing these variations can have a significant impact on reducing atmospheric pollution.

To address this research gap, this study took CH_4_ as the object and applies SPA to the EEIOA framework to examine the intricate sectoral relationships along supply chains. We conducted an in-depth analysis of the demand-driven mechanism of embodied CH_4_ emissions in China over a time series from 2000 to 2020, as well as the evolution characteristics of the supply chain path. The study considers the entire flow of demand-driven CH_4_ emissions from the production side, through intermediate emissions, to the consumption side, taking into account the characteristics and changes in demand, emission sectors and critical supply chain paths. Our findings serve as a fundamental cornerstone for the country’s efforts to establish effective national and local policies for CH_4_ mitigation.

## Methodology and data sources

### Environmentally extended input–output analysis (EEIOA)

The Environmentally Extended Input–Output Analysis (EEIOA) method can link the consumption of terminal goods and services with the CH_4_ emissions of various sectors in the supply chain. The IO model of $p$ sectors and $q$ types of final demand is:

1 $$\sum\limits_{{j = 1}}^{p} {z_{{ij}} + \sum\limits_{{k = 1}}^{q} {y_{i}^{k} } } = x_{i}$$

$z_{ij}$ is the intermediate input provided by sector i to j, $y_{i}^{k}$ is the final demand k of sector i, $x_{i}$ is the total output of sector i.

The basic row balance for a national-scale input–output table of China’s industry sectors written in matrix form can be expressed as:

2 $$AX + F - X^{m} = X$$

Herein, $${\text{A}} = \left[ {\begin{array}{*{20}c} {a_{11} } & {a_{12} } & {...} & {a_{1n} } \\ {a_{21} } & {a_{22} } & {...} & {a_{2n} } \\ \vdots & \vdots & \ddots & \vdots \\ {a_{n1} } & {a_{n2} } & {...} & {a_{nn} } \\ \end{array} } \right]$$ is the inter-industry requirements matrix.$AX$ represents the intermediate input. Specifically,$A$ represents the technology coefficients matrix, describing the relationship between each industry sector from the production process. $F$ represents the final demand vector categorised into six categories: rural consumption, urban consumption, government consumption, capital formation, stock increase and exportation. $X^{m}$ represents imports,$X$ represents the total output. The input–output (IO) table of China requires pre-processing before calculations. For this study, we used a time series competitive IO table, assuming that imports are produced locally and are distributed to intermediate use and final demand in the same proportion. As this study focuses on the reflection of supply chain of embodied CH_4_ emissions in China, the import items are excluded to isolate the domestic supply chain. We assume that the import proportions of each industry sector and the final demand are identical, referring to previous studies^34,35^. The proportion is calculated as follows:

3 $$m_{{_{ii} }} = \frac{{X_{i}^{m} }}{{X_{i} + X_{i}^{m} - f_{i}^{e} }}$$

4 $$A^{d} = (I - M)A$$

Herein,$M_{ii}$ is the share of imports in the supply of products and service to each industry sector and $A^{d}$ is the direct requirement of domestic production coefficients matrix. The new balance equations can be rewritten as:

5 $$X = Z^{d} + f^{d} + f^{e} = A^{d} X + f^{d} + f^{e}$$

Herein, $Z^{d}$ is the domestic intermediate demands input matrix, $f^{d}$ is the final demands vector after the imported items have been removed and $f^{e}$ is the domestic exports vector.

Based on Eq. (6), the following equation can be derived:

6 $$X = \left( {I - A^{d} } \right)^{ - 1} \left( {f^{d} + f^{e} } \right) = L^{d} \left( {f^{d} + f^{e} } \right)$$

Herein, $I$ is the identity matrix and $L^{d} = \, \left( {I - A^{d} } \right)^{ - 1}$ is the domestic Leontief inverse matrix.

According to Eq. (6), coupled input–output with CH_4_ emission data, the embodied CH_4_ emissions from the domestic final demand side (EEF) can be expressed as

7 $$EEF =\upvarepsilon ^{d} l^{d} \left( {f^{d} + f^{e} } \right) =\upvarepsilon \left( {f^{d} + f^{e} } \right) =\upvarepsilon f^{d} +\upvarepsilon f^{e}$$

Herein, $\varepsilon^{d}$ is the direct CH_4_ emission intensity, which equals the direct CH_4_ from the production side divided by the total output, $\varepsilon$ represents the embodied CH_4_ emissions intensity (i.e. direct plus indirect), $\varepsilon f^{d}$ is the domestic CH_4_ emissions embodied in consumption and $\varepsilon f^{e}$ is the CH_4_ emissions embodied in exports.

### Structural path analysis (SPA)

The SPA method is adopted to investigate the embodied CH_4_ emissions within China’s supply chain based on the input–output method. The domestic Leontief inverse matrix $L^{d}$ in Eq. (6) can be expanded using Taylor series approximation^27,34^, and its expansion can be expressed as:

8 $$L^{d} = \left( {I - A^{d} } \right)^{ - 1} = I + A^{d} + \left( {A^{d} } \right)^{2} + \left( {A^{d} } \right)^{3} + \cdots + \left( {A^{d} } \right)^{t} ,\mathop {\lim }\limits_{t \to \infty } (A^{d} )^{t} = 0$$

Each item on the right-hand side of Eq. (8) represents a different production layer (PL); a PL is defined as each element in the power series expansion. In this study, the $t$th production layer $PL^{t}$ is $\left( {A^{d} } \right)^{t}$ in the aforementioned equation. This equation represents the intermediate products purchased by the $t + 1$ production layer when the production layer $t$ is in production. The embodied CH_4_ emissions of final demand can be calculated by:

9 $$f^{d} = \left( {I - A^{d} } \right)^{ - 1} y^{d} = f^{d} ly^{d} + f^{d} A^{d} y^{d} + f^{d} \left( {A^{d} } \right)^{2} y^{d} + f^{d} \left( {A^{d} } \right)^{3} y^{d} + \cdots + f^{d} \left( {A^{d} } \right)^{t} y^{d}$$

Herein, $f^{d} \left( {A^{d} } \right)^{t} y^{d}$ denotes the CH_4_ emissions occurring at the $t$th production layer $\left( {PL^{t} } \right)$.

**Figure 1:**
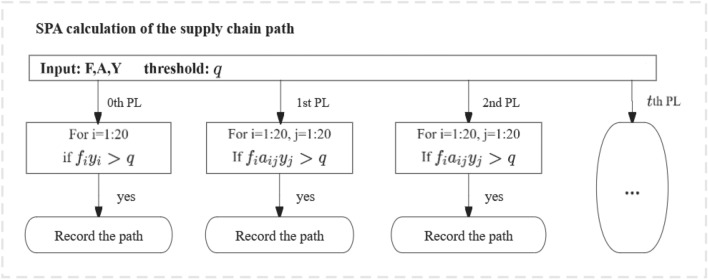
The computational process of the SPA method.

The production layer is a virtual concept based on the supply chain, and each production layer is distributed among the 20 industry sectors. Considering the CH_4_ emission in the whole process of automobile production as an example, where $y^{d}$ is the production demand for an automobile, $f^{d} Iy^{d}$ represents the CH_4_ directly emitted by the automobile manufacturer. As companies that manufacture automobiles require upstream industry sectors to supply them with parts and components, the production of such inputs result in CH_4_ emissions amounting to $f^{d} A^{d} y^{d}$. However, the production of the parts and components requires the inputs $\left( {A^{d} } \right)^{2} y^{d}$ from their upstream industry sectors, which may cause $f^{d} \left( {A^{d} } \right)^{2} y^{d}$ CH_4_ emissions. Thus, the whole process continues through the infinite extension of the power series. By analogy, for the supply chain path “x → y → z → final demand”, sector z is in the zeros production layer, sector y is in the first production layer and sector x is in the second production layer. The longer the supply chain, the more production layers it occupies, with all 20 industry sectors distributed across each layer, and the proportion of each sector in each production layer is different, reflecting the structure of the supply chain. We summarize the computational process of SPA method in Fig. 1.

### Data source and processing

This study required two sets of data: China’s IO table (NBSC, 2000, 2002, 2005, 2007, 2010, 2012, 2015, 2017; 2020) as well as CH_4_ emissions inventories^36^. As the industry sectors of the input–output tables vary from year to year, this study first merged the industry sectors of years 2002, 2007, 2012, 2017 and 2020 into 42 sectors. Subsequently, we consolidated the 40 or 42 sectors from 2000 to 2020 into 20 unified sectors (see Appendix Table S1). To maintain consistency of the 20-year time series, the latest CH_4_ emission data from the Emission Database for Global Atmospheric Research (EDGAR v7.0) were used based on IPCC 2019 guidelines^37,38^. For consistency, we further consolidated CH_4_ emissions inventories into 20 aggregated industry sectors. Referring to related research, this study attributes one-third of CH_4_ emissions from solid waste disposal, incineration and open burning of waste and wastewater treatment and discharge sectors in EDGAR v7.0 to Sector 20 (commercial and public services activities). Additionally, depending on the proportion of the total output of the IO table in the corresponding year, sectors with significant coverage in EDGAR v7.0 were divided into several small sectors in the inventory^13,39^.

## Results and discussions

### The embodied CH_4_ emission from both production and final demand during 2000–2020

**Figure 2:**
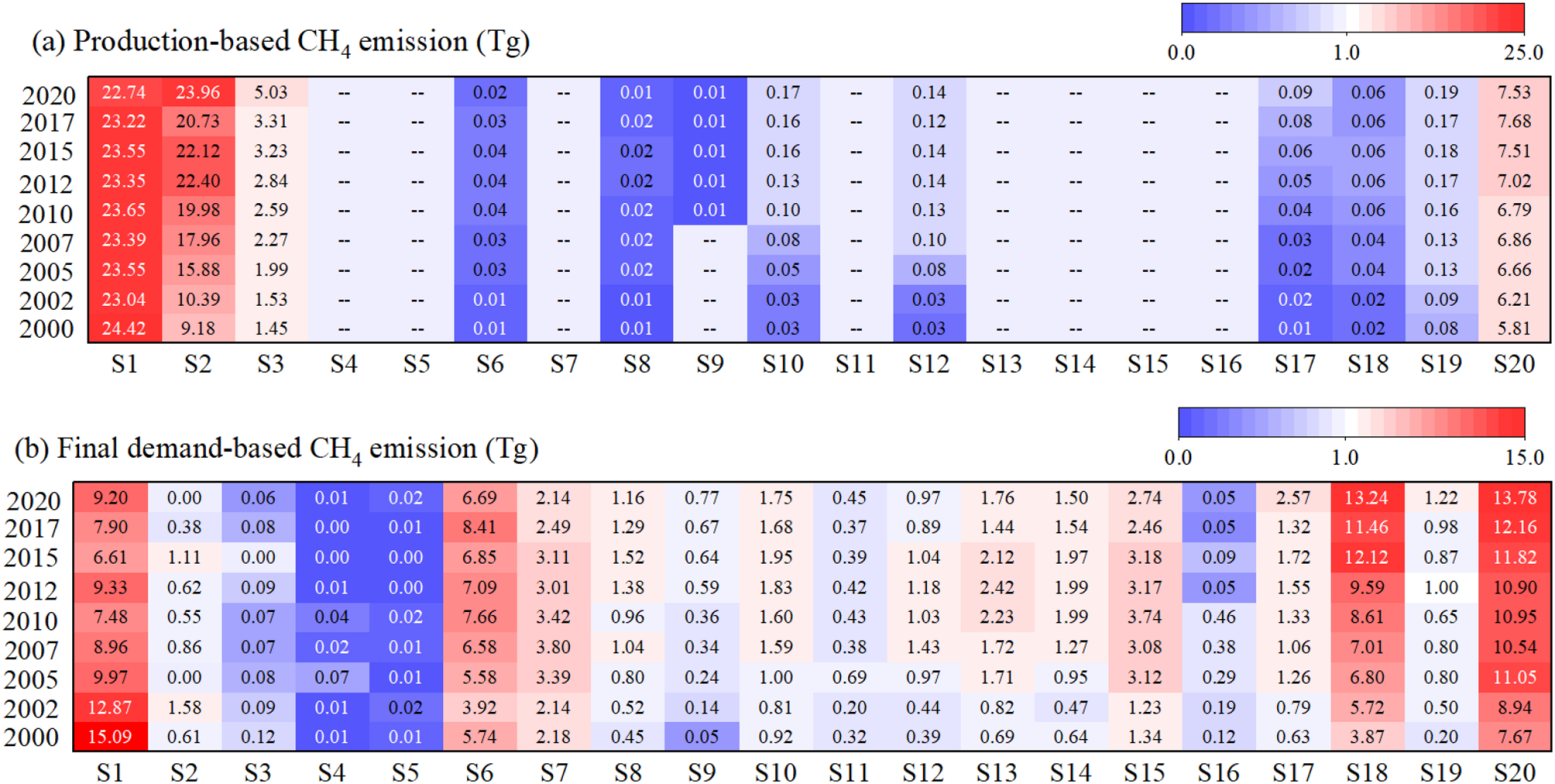
CH_4_ emissions in China based on sectoral production and final demand (2000–2020). Note: The horizontal axis shows sector codes which are specified in Appendix 1, and the vertical axis indicates the year.

**Figure 3:**
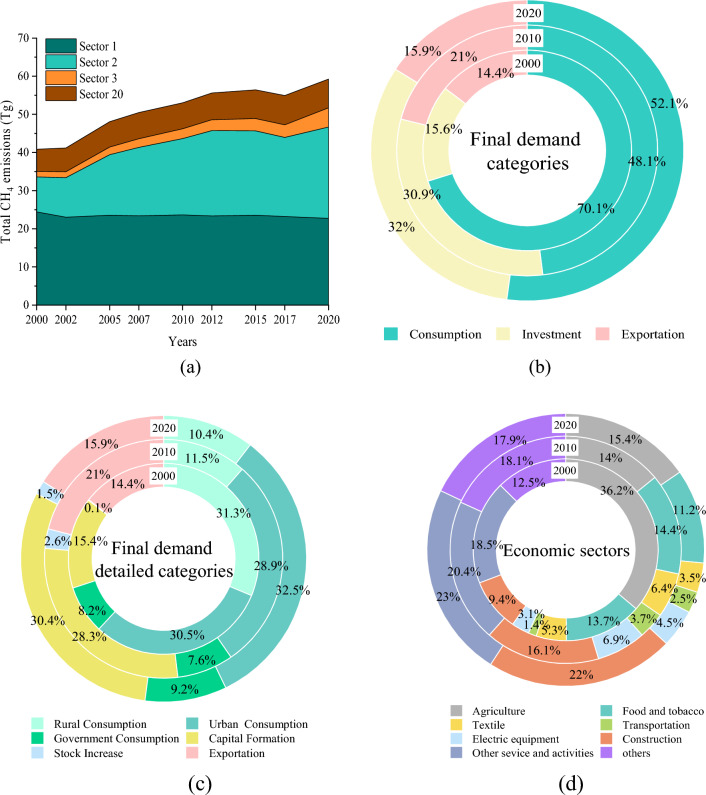
The composition of China’s embodied CH_4_ emissions in the economic system.

Production-based and consumption-based CH_4_ emissions for 20 sectors in China in 2000, 2002, 2005, 2007, 2010, 2012, 2015, 2017 and 2020 are presented in Fig. 2. From the production perspective (Fig. 2a), China’s total CH_4_ emissions exhibited a growth trend, from 41.1 Tg in 2000 to 60.0 Tg in 2020, with a growth rate of 46.0% (As shown in Fig. 3a). According to critical emission sectors from the production side, agricultural activities were the primary contributors to CH_4_ emissions, with the agricultural sector (S1) accounting for approximately half of the total emissions in 2000 and 2010 (59.5% and 44.2%, respectively), but declining to 37.9% in 2020. Coal mining (S2) consistently ranked second in terms of CH_4_ emissions, increasing from 22.4% in 2000 to 37.3% in 2010 and reaching 40.0% in 2020. Petroleum and natural gas (S3) along with other service and activities (S20) lagged behind. In addition to the mentioned four major emission sectors, chemical industry (S10), non-ferrous metals (S12) and transport and postal service (S19) also contributed to a smaller amount of CH_4_ emissions. All other sectors continued to experience slight increases in emissions.

Depending on whether the emissions were measured from a production or consumption perspective (Fig. 2b), the dominant sectors are remarkably distinct. From the consumption perspective, the embodied CH_4_ emission in agriculture (S1), food and tobacco (S6), construction (S18) and other service and activities (S20) held the top four positions in final demand. These four sectors contributed 32.4 Tg, 34.7 Tg and 42.9 Tg emissions, respectively, representing 78.8%, 64.8% and 71.6% of total emissions in 2000, 2010 and 2020. From 2000 to 2020, the share of embodied CH_4_ emissions in agriculture (S1) declined in the first 10 years and then fluctuated in the last 10 years, while other service and activities (S20) grew rapidly in the first 5 years, but the growth rate slowed down after 2007. The construction sector (S18) exhibited the fastest growth, generating more embodied CH_4_ emissions in China due to increased construction activity, while the food and tobacco sector (S6) exhibited minor fluctuations.

Embodied CH_4_ emissions represent the sum of all CH_4_ emissions from the production of products or services, including direct emissions and indirect emissions. For example, in the construction industry, the direct CH_4_ emissions during the production activities of this sector are minimal, but the production and transportation of various building materials required in the production process will generate substantial CH_4_ emissions. Thus, the embodied CH_4_ emissions of the construction sector are notable. However, the coal, oil and natural gas production sectors exhibit an opposite trend. The production processes involved in these sectors will cause a large amount of CH_4_ emissions and mainly supply energy products for other sectors.

Figure 3 presents the changes in the composition of China's embodied CH_4_ emissions in the economic system between 2000 and 2020 in terms of final demand categories and industry sectors. Consumption, investment, as well as exportation were the three driving forces to stimulate economic growth in China’s society^40^. As illustrated in Fig. 3a, consumption contributed the largest fraction of 70.1%, 48.1% and 52.1% to the total emissions for the years 2000, 2010 and 2020 (i.e., 28.8 Tg, 25.8 Tg and 31.2 Tg), respectively, followed by investment and export. As shown in Fig. 3b, in the consumption-driven category, rural consumption, urban consumption and government consumption were included. In 2000, urban consumption was associated with fewer embodied CH_4_ emissions than rural consumption. Thereafter, the emissions of urban consumption have gradually exceeded those of rural consumption, and the gap between the two categories has continued to widen. In 2020, emissions generated by urban consumption were 3.1 times greater than emissions generated by rural consumption.

The investment-driven category comprises of capital formation and stock gains. China’s economic development has been largely driven by investment activities over the long run, with approximately 90.0% of the investment-driven embodied emissions generated from capital formation, whereas the remaining emissions were attributed to stock increases. Substantial direct and indirect CH_4_ emissions during the production process were driven by the real estate industry and infrastructure construction. As shown in Table S2, the total embodied CH_4_ emissions driven by China’s investment activities in 2020 were three times higher than those in 2000 (6.4–19.2 Tg).

In 2000, exports contributed 5.9 Tg of embodied CH_4_ emissions, accounting for approximately 14.4% of total embodied CH_4_ emissions. Embodied CH_4_ emissions associated with exports increased from 2000 to 2007 (5.9–12.9 Tg) but decreased from 2010 to 2020 (11.2–9.6 Tg). The major contributors of embodied CH_4_ emissions were manufacturing sectors that produced major export products, including textiles, timber and paper products, chemical products and electric equipment.

As illustrated in Fig. 3c, agriculture, food and tobacco, textile, transportation, electric equipment, construction and other services and activities are the major seven sectors with the highest embodied CH_4_ emissions from 2000 to 2020. To reduce the complexity of the economic system, we picked the top seven sectors with highest emissions and merged the other sectors into another one. Over 80% of the total embodied emissions are attributed to these seven sectors. In particular, emissions from agricultural sector have decreased significantly. In 2000, the agricultural sector contributed 15.1 Tg emissions (40.3% of the total emission), ranking first among the four sectors in terms of embodied emissions, and its proportion has declined ever since. The other services and activities sector has grown annually, surpassing agriculture, to become the largest emitter since 2010. Moreover, there has been a steady increase in the share of the construction sector. However, the emissions from food and tobacco, textiles, transportation and electric equipment did not change significantly.

### Embodied CH_4_ emissions in different production layers during 2000–2020

We analysed the embodied CH_4_ emission from various industry sectors from 2000 to 2020. Drawing on related literature^15,29^, we address an important question: what are the sources of direct CH_4_ emission involved in the production of final products within particular sectors? How can we trace the initial CH_4_ emissions from final consumption back to intermediate consumption processes? To address these gaps in the literature, the SPA method has been used to explore the supply chain path of CH_4_ emission among different industry sectors in China. Additionally, the Sankey diagram has been utilized to map emission flows throughout the entire supply chain, helping determine the origins (consumption attribution) and destinations of emissions (production attribution) embodied in final products.

**Figure 4:**
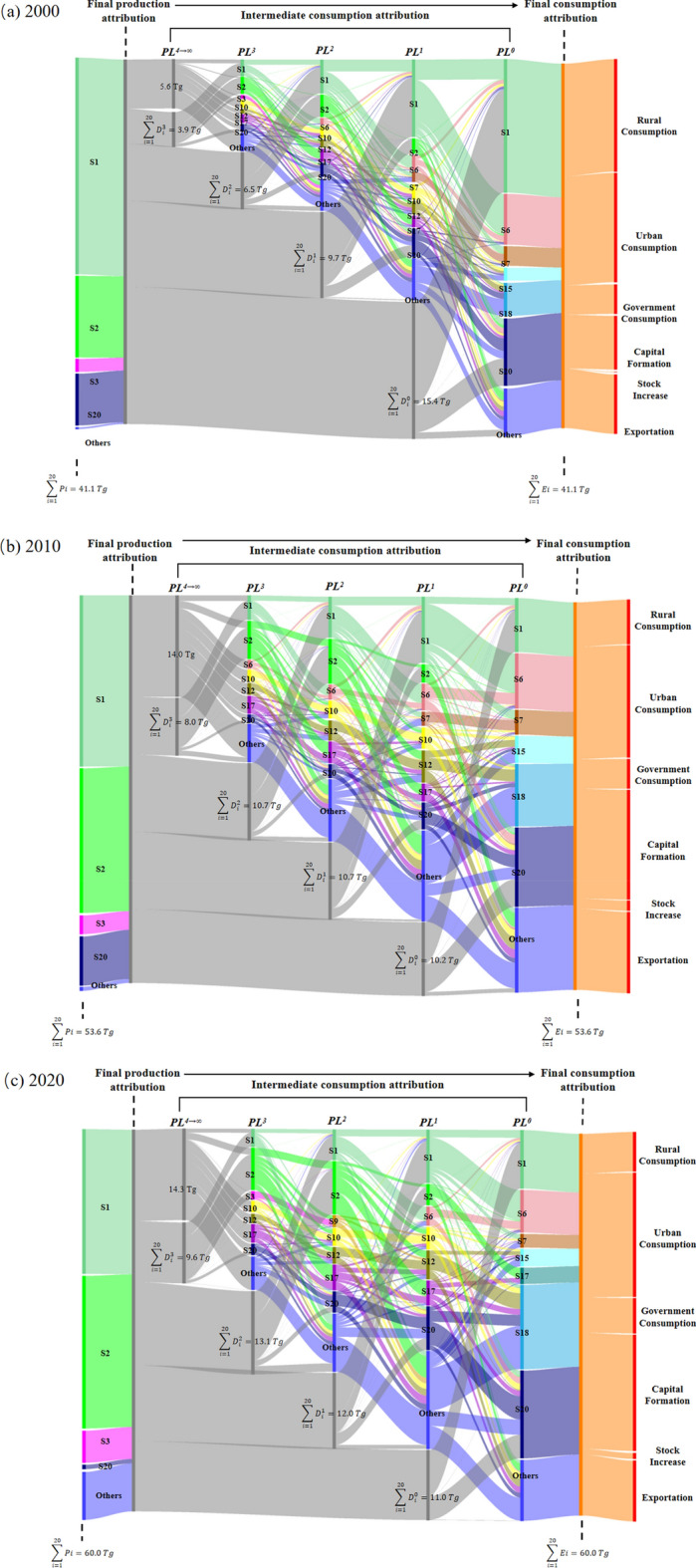
Sankey diagrams of China’s embodied CH_4_ emission flows in (**a**) 2000; (**b**) 2010; (**c**) 2020. Note: As shown in the diagram from left to right, each sector is represented by a different colour. The diagram reveals the transfer of embodied CH_4_ emissions from the final production sectors via intermediate consumption to the sectors of final consumption, and ultimately to the final demand. Final production attribution shows the direct emissions occurring in the production processes. PL^4^ and higher layers are combined to provide a comprehensive view. The central part of the diagram presents the intermediate consumption attributions for each aggregated sector at PL^1^, PL^2^, and PL^3^, which indicated by the gray “flows” linking back to the final production attribution. The colourful “flows” from PL^t^ to PL^t−1^ measure embodied CH_4_ emissions in the output of the sector at PL^t^ purchased by the sector at PL^t−1^. ${\text{D}}_{\text{s}}^{\text{t}}$ represents CH_4_ emissions from sector s at PL^t^. The orange “flows” on the right side indicate the 20 aggregated sectors’ embodied emissions attributed to final demand. The sum of CH_4_ emissions in final production equals those in final consumption. Detailed sectoral information are provided in Appendix table S1.

Figure 4 illustrates the embodied CH_4_ emission flows throughout the supply chain driven by final demand in a Sankey diagram at tier 0, tier 1 and higher tiers (tier 2, tier 3, and tier 4 → $\infty$), which lists the embodied CH_4_ emissions of various industry sectors in each production layer in 2000, 2010 and 2020, respectively. In this study, the embodied CH_4_ emissions of the 0th production layer (PL^0^) represents the CH_4_ directly emitted by the 20 industry sectors in the 0th production layer to meet the final demand. The embodied CH_4_ emissions of the first production layer (PL^1^) represent all CH_4_ emissions from 20 industry sectors in the first production layer providing intermediate products for the final product of the sector. Moreover, the embodied CH_4_ emissions of the 2nd production layer (PL^2^) represent all direct CH_4_ emissions from 20 industry sectors in the second production layer providing intermediate products for the final product of the sector.

Appendix Table S3 shows the distribution of the embodied CH_4_ emissions of various industry sectors in different production layers of the supply chain in 2000, 2010 and 2020. The agriculture (S1) sector exhibited the highest total embodied CH_4_ emissions in 2000, whereas other services and activities (S20) surpassed agriculture (S1) and ranked first after 2010. Most of the embodied CH_4_ emissions of agriculture (S1), coal mining (S2), petroleum and nature gas (S3) and other services and activities (S20) are distributed in PL^0^. The common feature of the distribution of embodied CH_4_ emissions in most sectors of the manufacturing industry (e.g., S4–S8, S10–S13 and S16) is that the emissions from PL^0^ are minimal, and most of them are concentrated in PL^1^, PL^2^ or even higher levels. Petroleum processing (S9) and gas production and supply (S17) have the highest proportion of emissions in PL^1^, while the other services and activities sector (S20) exhibits an even distribution between PL^1^ and PL^2^. Both transportation (S14) and electric equipment (S15) at PL^4→∞^ accounted for more than 40.0% of the emission proportion. From 2000 to 2020, the distribution of embodied CH_4_ emissions in most sectors was concentrated in the upper production layers.

From the consumption-based perspective, urban consumption, capital formation, export and rural consumption emerged as the major final demand categories of CH_4_ emissions, accounting for 32.5%, 30.4%, 15.9% and 10.4% of the national total in 2020, respectively. The embodied CH_4_ emissions from PL^t^ induced by final demand can be traced to 20 sectors. As shown in Fig. 4, from the right to the left of the image, the initial CH_4_ emission source can be traced from the final consumption side through intermediate consumption links. For instance, agriculture (S1), food and tobacco (S6), construction (S18) and other service and activities (S20) at PL^0^ provided the primary source to meet the final demand directly from 2000 to 2020. In the PL^0^, construction (S18) produced an increasing amount of CH_4_ emissions in recent years, accounting for 9.4% emissions in 2000, rising up to 22.1% in 2020. However, no emissions were observed in this sector in other production layers, indicating that the products of construction (S18) were mainly used as final products to directly meet the final demand. As reported in the middle section of the figure, in each production layer, agriculture (S1) accounted for the largest share of embodied CH_4_ emissions among the 20 sectors, driving 15.1, 7.5 and 9.2 total embodied CH_4_ emissions in 2000, 2010 and 2020, which accounted for 36.7%, 14.0% and 15.3% of the total embodied emissions of those years, respectively. Other service and activities (S20) also accounted for a large proportion of emissions at all production layers. This sector drove 18.7%, 20.4% and 23.0% of the total embodied CH_4_ emissions in 2000, 2010 and 2020, respectively. The industry sectors with prominent emissions in the intermediate production layers were the chemical industry (S10), non-ferrous metals (S12) and electric power and heat production and supply (S17). However, these sectors do not emit prominently in the PL^0^, indicating that the manufacturing industry occupied an important position in the intermediate production links of embodied CH_4_ emissions. From the perspective of production attribution, agriculture (S1) and coal mining (S2) consistently dominated CH_4_ emissions, together contributing more than three-quarters of the national total emissions in 2000, 2010 and 2020 (81.8%, 81.5% and 77.9%), respectively. Petroleum and natural gas (S3) and other service and activities (S20) were responsible for another quarter of the CH_4_ emissions.

**Figure 5:**
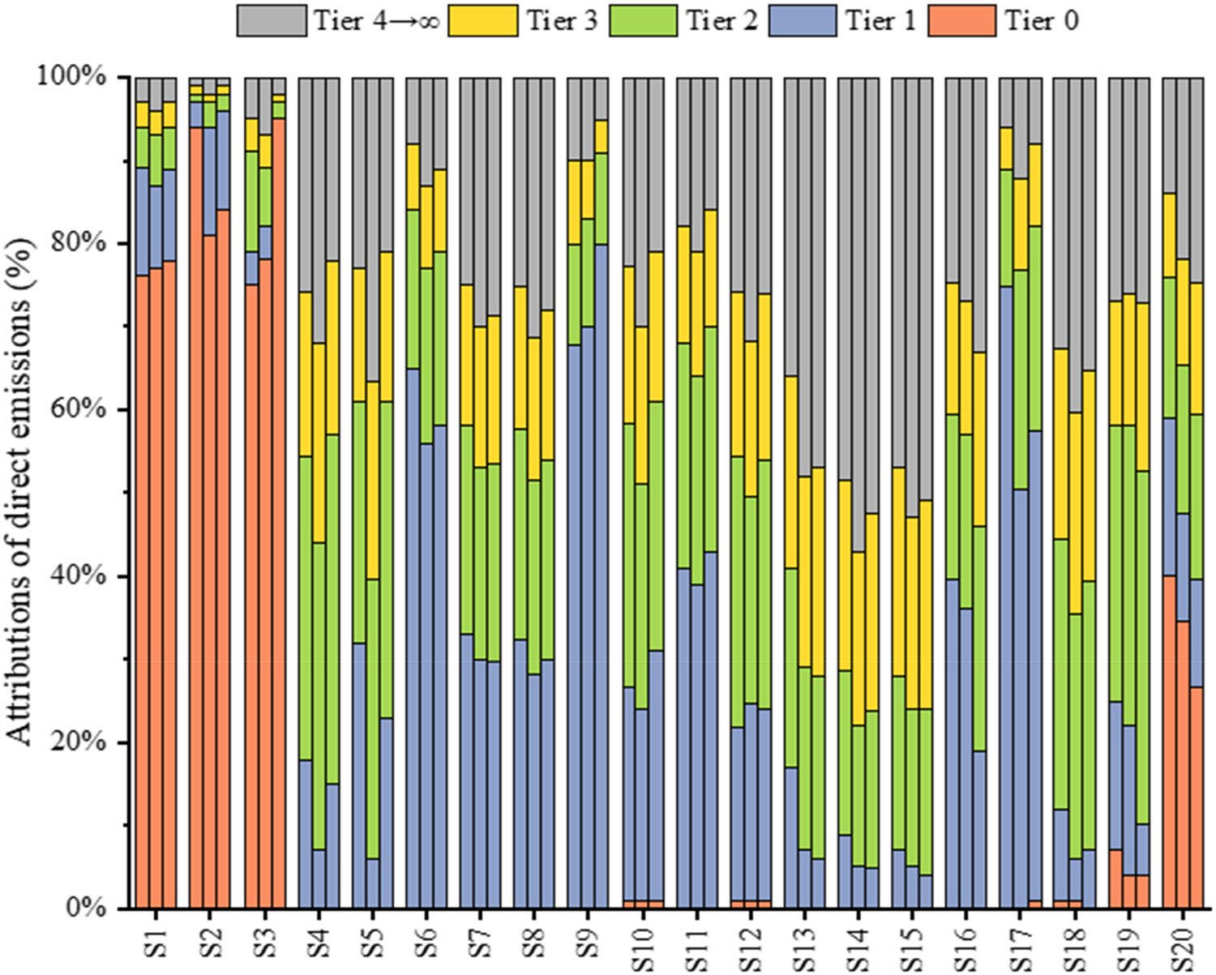
Distributions of direct CH_4_ emissions in production layers along supply chains from 20 sectors (From left to right, each group is 2000, 2010 and 2020).

Figure 5 shows the distribution of embodied CH_4_ emissions in each production layer of 20 industry sectors summarised in this study from 2000 to 2020. Agriculture (S1), coal mining (S2) and petroleum and nature gas (S3) share a similar distribution structure, with 80% of their emissions distributed to PL^0^. Around 70% of petroleum processing (S9) emissions are concentrated in PL^1^. The emissions of PL^0^ from the manufacturing (S4–S8, S10–S13 and S16), electric (S15 and S17) and construction (S18) industries are almost non-existent, and the major emissions emerge from the PL^1^ and higher tiers, especially for the construction sector, which has a high proportion of emissions from PL^4^ and higher levels (32.5–35.2%). Within the transportation sectors, including S14 and S19, more than 70% of emissions originate from PL^2^ or higher levels. S19 exhibits decreasing PL^0^ emissions (7.0–4.0%), whereas S14 has almost no PL^0^ emissions. The main emissions of other service and activities (S20) originate from PL^0^, and the distribution of other layers over the years is relatively balanced.

### Supply chain path analysis of embodied CH_4_ emissions during 2000–2020

To identify the manner in which industry sectors drive emissions in each layer, we extracted and ranked each critical supply chain, starting with production, to intermediate consumption and finally ending with final consumption. As listed in Table 1, we analysed the top 20 ranking paths through which final demands drove embodied CH_4_ emissions in various sectors. During the 20-year period, total path value in the top 20 supply chains decreased by 8.3% from 21.7 Tg in 2000 to 20.1 Tg in 2000, and they are all concentrated in PL^0^, PL^1^ and PL^2^.

**Table 1:** Top 20 ranking supply chain paths for embodied CH_4_ emissions from 2000 to 2020.

	Rank	Tier	Path value	Contribution	Path		Tier	Path value	Contribution	Path		Tier	Path value	Contribution	Path
**2000**	1	0	5.9	14.3%	S1 → RC	**2010**	1	2.7	5.0%	S1 → S6 → UC	**2020**	0	3.6	6.0%	S1 → UC
	2	0	4.2	10.1%	S1 → UC		0	2.1	4.0%	S1 → UC		1	2.6	4.3%	S1 → S6 → UC
	3	1	1.8	4.3%	S1 → S6 → UC		0	1.9	3.6%	S1 → RC		2	1.9	3.1%	S2 → S11 → S18 → CF
	4	1	1.5	3.6%	S1 → S6 → RC		0	1.4	2.7%	S20 → UC		0	1.8	3.0%	S1 → RC
	5	0	1.3	3.3%	S20 → GC		0	1.3	2.5%	S20 → GC		0	1.3	2.2%	S20 → GC
	6	1	0.9	2.2%	S1 → S1 → RC		2	1.3	2.4%	S2 → S11 → S18 → CF		0	1.3	2.2%	S20 → UC
	7	0	0.8	1.9%	S20 → UC		0	1.1	2.1%	S1 → CF		1	1.1	1.9%	S2 → S17 → UC
	8	0	0.7	1.6%	S1 → CF		1	1.0	1.9%	S1 → S6 → RC		0	0.9	1.5%	S1 → SI
	9	1	0.6	1.5%	S1 → S1 → UC		1	0.5	1.0%	S1 → S7 → Ex		1	0.9	1.5%	S1 → S6 → RC
	10	0	0.5	1.3%	S1 → Ex		2	0.5	1.0%	S1 → S6 → S6 → UC		2	0.5	0.9%	S2 → S12 → S18 → CF
	11	0	0.5	1.2%	S20 → RC		1	0.5	1.0%	S2 → S17 → UC		0	0.5	0.9%	S20 → CF
	12	0	0.4	1.0%	S2 → Ex		0	0.4	0.7%	S20 → CF		2	0.5	0.8%	S1 → S6 → S6 → UC
	13	1	0.4	0.9%	S1 → S6 → Ex		0	0.4	0.7%	S20 → Ex		2	0.5	0.8%	S2 → S17 → S18 → CF
	14	0	0.4	0.9%	S1 → SI		2	0.3	0.7%	S2 → S12 → S18 → CF		1	0.5	0.8%	S1 → S1 → UC
	15	1	0.4	0.9%	S1 → S7 → Ex		2	0.3	0.6%	S1 → S1 → S6 → UC		1	0.5	0.8%	S20 → S18 → CF
	16	1	0.3	0.9%	S2 → S17 → UC		0	0.3	0.6%	S20 → RC		1	0.4	0.6%	S20 → S20 → GC
	17	2	0.3	0.8%	S2 → S11 → S18 → CF		1	0.3	0.6%	S1 → S6 → Ex		3	0.4	0.6%	S2 → S11 → S11 → S18 → CF
	18	0	0.3	0.8%	S20 → Ex		0	0.3	0.6%	S1 → Ex		1	0.4	0.6%	S20 → S20 → UC
	19	0	0.3	0.7%	S2 → RC		1	0.3	0.5%	S1 → S1 → UC		2	0.3	0.6%	S1 → S1 → S6 → UC
	20	1	0.3	0.7%	S20 → S18 → CF		2	0.3	0.5%	S1 → S6 → S20 → UC		1	0.3	0.5%	S1 → S7 → Ex

Note: Urban Consumption, UC; Rural Consumption, RC; Government Consumption, GC; Capital Formation, CF; Stock Increase, SI; and Export, Ex. The unit of Path value is Tg. Sectoral information is provided in Table S1.

In 2000, the path of “Agriculture → Rural consumption” contributed the largest share of the total embodied CH_4_ emissions accounting for 14.3% among total path in 2000 (5.9 Tg). This path implied that rural consumption caused 5.9 Tg of embodied CH_4_ emissions using the product from the agricultural sector in 2000. Moreover, the path, “Other services and Activities → Construction → Capital formation”, contributed the lowest share, accounting for 0.7% of the total embodied CH_4_ emissions in the same year, reflecting that the development of service and construction sector had not been strongly supported at that stage. Furthermore, 11 of the top 20 supply chain paths involved the agricultural sector, which at that time was closely related to its production structure and consumption intensity. In addition, the improvement of living standards of agricultural workers was dependent on the extraction of a large number of resources.

Compared with 2000, the top ranked path transformed to “Agriculture → Food and tobacco → Urban consumption” in 2010. The top-ranked path of these two years was all related to the agricultural sector, in addition to linking to final demand, agriculture-based paths also provide products and services to other sectors, such as food and tobacco and textiles. In addition, “Coal mining → Non-metalmineral products → Construction → Capital formation” appeared in the top 10 supply chain paths for the first time, reflecting the rising contribution of the construction industry. As a result of China’s growing urbanisation rate during the study period, the urbanisation rate increased from 39.2% in 2000 to 50% in 2010, and the total number of construction enterprise increased from 47,518 in 2000 to 71,863 in 2010.^36^ Thus, the rapid development of construction inevitably drove the production of non-metal mineral products from the coal mining industry.

Similarly, the path “Agriculture → Urban consumption” ranked the largest in terms of emissions in 2020. Arguably, this change reflects the increasing gap between urban and rural consumption in terms of embodied CH_4_ emissions over the past two decades, which is associated with China’s 13th Five Year Plan^41^ to promote agricultural modernisation and accelerate agricultural structural adjustment. In total 6 of the 20 supply chain paths are rooted in other service and activities sector, indicating the Chinese government has begun to pay attention to the service sector.

From 2000 to 2020, the number of supply chain paths driven by urban consumption (5-8-8) and capital formation (3-4-6) illustrated a trend of rapid growth, and especially after, 2010. Over one-third of the national CH_4_ emissions were embodied in the final demand for products from the top 20 supply chain paths from 2000 to 2020, primarily driven by urban consumption and capital formation. 8 out of 20 paths were driven by urban consumption. In contrast, the number of paths driven by rural consumption (5-3-2) and exportation (5-4-1) declined gradually. Moreover, the number of paths driven by government consumption (1-1-2) and stock increase (1-0-1) was relatively low but stable.

**Figure 6:**
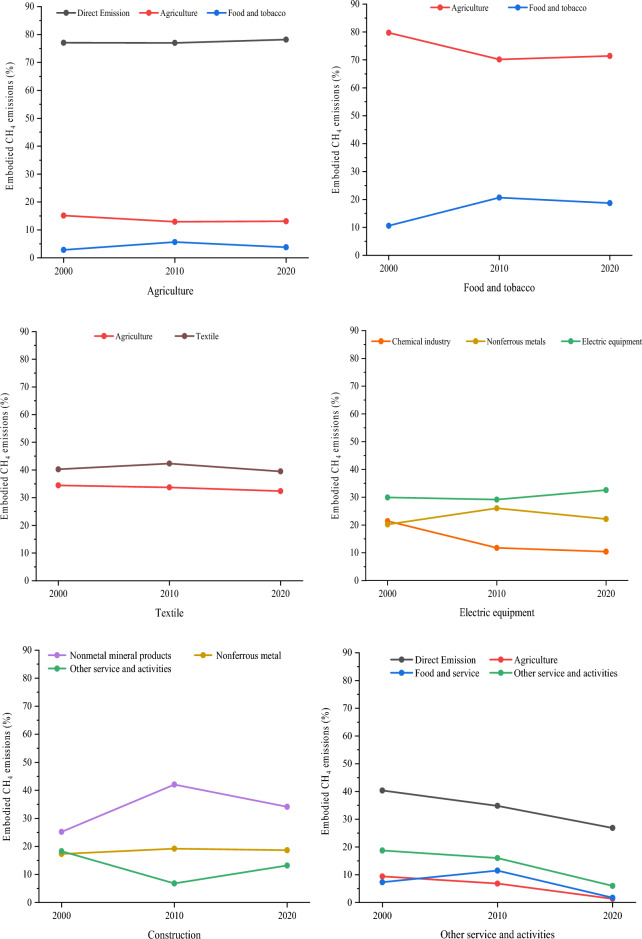
Changes in embodied CH_4_ emissions from PL^1^ purchased by main consumption-side sectors.

Figure 6 presents the six major sectors with the highest embodied CH_4_ emissions on the consumption side between 2000 and 2020. Overall, the proportion of inputs purchased from PL^1^ by major sectors has changed significantly, whereas the sector type has remained relatively stable. For agricultural sector (S1), approximately 75% of emissions are direct emissions, with a decrease in upstream agricultural emissions (15.2–13.1%) and an increase in emissions from the food and tobacco sector (S6) (2.9–3.8%). Over 90% of the embodied CH_4_ emissions of the food and tobacco sector (S6) originate from both the sector itself and the agricultural sector at PL^1^. The embodied CH_4_ emissions from the textile sector (S7) corresponding to PL^1^ are mainly concentrated in the textile and agricultural sector. Electric equipment emerged as the most significant component of inputs purchased by the electric equipment sector (S15) from PL^1^, which grew from 29.9% in 2000 to 32.6% in 2020. Nevertheless, the embodied CH_4_ emissions resulting from the production of intermediate products of chemical industry purchased from PL^1^ is decreasing (21.4–10.4%). In the construction industry (S18), the final demand of the industry drives the increase in emissions from its upstream material sectors including non-metal mineral products (25.2–34.1%) and non-ferrous metal (17.3–18.7%). Moreover, the direct emissions of other service and activities sector (S20) in PL^0^ declined significantly (40.4–26.9%), and the three other sectors (S1, S6 and S20) purchased from PL^1^ also exhibit a decreasing trend.

The 14th Five Year Plan period holds great significance for China's climate change efforts. As a crucial part of addressing climate change, CH_4_ emission reduction has also ushered in historic development opportunities. Understanding the evolution characteristics of the supply chain path of CH_4_ emissions is crucial for policy makers to consider the effects of supply chains on resources and the environment. Table S4 summarises the literature analysing the supply chain of embodied CH_4_ emissions on a national scale. Existing studies have analysed the CH_4_ reduction within the context of overall GHG reduction and calculated this parameter from a single-year perspective^35,42^. In contrast, our findings explore the dynamic variation of China’s embodied CH_4_ emissions on the key sectors and supply chain paths over a 20-year period, which could inform future CH_4_ reduction policies. From the key sector perspective, the top three sectors from the final demand side of this study are S1 (agriculture), S6 (food and tobacco) and S20 (other service and activities) in 2012, which are consistent with the results reported by Zhang et al.^12^. However, upon tracking the dynamic change of the key sectors, we observe a shift in the top three sectors in 2020, now comprising S1 (agriculture), S18 (construction) and S20 (other service and activities). From the perspective of key supply chain path, our findings align with those of Zhang et al.^12^ and Zhang et al.^35^ in identifying “Farming, Forestry, Animal Production and Fishery → Urban Consumption” as the top-ranked path in 2012. Moreover, our results indicate that the top-ranked path after 2010 also starts from S1 (agriculture) and ends in urban consumption.

Traditionally, emission reduction approaches focus on the responsibility of emitters for reducing emissions, primarily focusing on reducing production-related direct emissions. Most of the production-side CH_4_ emissions come from agricultural and energy related activities. Although reduction of direct CH_4_ emission is an important objective, it is essential to recognise that emission responsibilities should not solely rest on the production side. Throughout the supply chain, emissions resulting from final demand-driven consumption and intermediate production processes also significantly contribute to CH_4_ reduction efforts.

Studies based on input–output performance analysis have shown that the coal mining industry consistently ranks high in embodied CH_4_ emissions from both production and consumption perspectives. In 2020, 5 of the top 20 paths for embodied CH_4_ emissions can be traced back to coal mining (S2). As China is the world’s largest coal producer^43^, a large amount of CH_4_ adsorbed in coal escapes into the atmosphere during the entire process of mining, surface treatment, coal preparation, transportation and terminal utilisation. Thus, China can control CH_4_ emissions by optimising its energy consumption structure, utilising cleaner power generation technologies, developing a reasonable plan for CH_4_ emissions reduction for abandoned coal mines and developing new energy industries vigorously. The critical supply chain paths identified in this study go beyond illustrating the production and consumption perspectives. They emphasise the importance of considering the embodied CH_4_ emissions flowing between industry sectors. For example, the CH_4_ emission path, driven by consumption through the intermediate products of the food and tobacco sector (S6) and traced back to the agricultural sector (S1) at the production side, also frequently appears in the top-ranked path. This result implies that the embodied CH_4_ emissions flowing between the agricultural and the food industries cannot be ignored. Moreover, it is crucial to control CH_4_ emissions from upstream sectors driven by the construction industry. The construction sector (S18) has exhibited the fastest growth rate since 2000. The ranking of the path ‘Coal Mining (S2) → Non-metal Mineral Products (S11) → Construction (S18) → Capital Formation’ has risen from 17th position in 2000 to the 6th position in 2010 and reached the 3rd position in 2020. Furthermore, among the top 20 embodied CH_4_ emission paths, those with the construction sector as the final product sector are all driven by capital formation, indicating that the construction sector, particularly infrastructure construction and real estate, has stimulated the development of energy-intensive industries such as non-metallic materials and metal materials. Although optimising China’s industrial structure and reducing the dependence on China’s economy on investment are crucial for directly reducing CH_4_ emissions, its indirect emission reduction effects are also worth noting. Therefore, it is vital to reduce the CH_4_ emissions attributed to each sector involved in the production and consumption sides of the supply chain for improving the efficiency of coordinated emission reduction between sectors.

## Conclusion and policy implications

Based on the EEIOA and SPA models, this study measured and analysed the supply chain of China’s embodied CH_4_ emissions from 2000 to 2020, focusing on key sectors, final demand, production layers and supply chain paths. The following key conclusions were drawn based on the findings: Over the past two decades, the total path value of the top 20 supply chains decreased. In most sectors, the distribution of emissions from PL^0^ was low, and most of the emissions were concentrated in PL^1^, PL^2^ and in even higher levels. There was an increase in supply chain paths driven by urban consumption and capital formation, a decrease in supply chain paths driven by rural consumption and export and a stable number of supply chain paths driven by government consumption and stock increase. The top-ranked supply chain path of embodied CH_4_ emissions tended to change from (starting from production to consumption) “Agriculture → Rural consumption” in 2000 to “Agriculture → Food and tobacco → Urban consumption” in 2010 to “Agriculture → Urban consumption” in 2020. This change can be partially attributed to the increasing gap between urban and rural consumption. As China’s economy develops rapidly, CH_4_ emissions will rise further. These results highlight the significance of reducing CH_4_ emissions in China, as they provide a platform for monitoring the dynamic changes in the country’s total anthropogenic CH_4_ emissions over time. Therefore, clear reduction targets for CH_4_ emissions in key industries and controlling supply chain pathways are crucial strategies for realizing China’s 2060 carbon neutrality goal while maintaining economic development.

The short life cycle of CH_4_ in the atmosphere makes stabilising CH_4_ emissions a powerful tool for controlling air pollutants. The results of embodied CH_4_ emissions throughout the entire supply chain yield essential information for Chinese policy makers to identify key areas for action. First, it is recommended to increase efforts to reduce CH_4_ emissions in the coal mining industry. As previously mentioned, coal mining generates substantial CH_4_ emissions that are increasing over time. In 2020, 5 of the top 20 paths for embodied CH_4_ emissions can be traced back to coal mining activities. Therefore, reducing coal mining emissions or replacing coal with cleaner energy is necessary to reduce CH_4_ emissions at the national level. Second, it is necessary to control the emissions of CH_4_ from upstream sectors driven by the construction industry. The construction industry exhibited the fastest growth rate since 2000, and all supply chain path related to construction sector were all driven by capital formation. This finding suggests that a large amount of investment flows into the construction sector represented by infrastructure construction and the real estate industry. Thus, if the government can reasonably control the construction activities and avoid overinvestment and redundant construction, then CH_4_ emissions from the construction sector can be greatly reduced. Third, the embodied CH_4_ emissions flowing between the agricultural and food and tobacco industry are significant and cannot be ignored. From either the production or consumption perspectives, agricultural sector emissions consistently rank among the top three positions. The CH_4_ emission path, driven by urban consumption and traced back to the agricultural sector at the production end through intermediate products in the food and tobacco industry, also frequently appears in the top ranked paths. Thus, livestock and poultry farms should be scaled and standardised, rice should be irrigated intermittently, organic fertilisers should be fully fermented, and dietary consumption structures should be balanced and diversified to reduce the embodied CH_4_ emissions associated with agriculture, food and tobacco production.

## Supplementary Information

Supplementary Tables.

## Data Availability

Data is provided within the manuscript or supplementary information files.
