# Supplementary material for: The temporal variation of CH4 emissions embodied in Chinese supply chains, 2000–2020
Source: Sci Rep. 2024 May 29;14:12379. doi: 10.1038/s41598-024-62979-z (PMC11637025; doi:10.1038/s41598-024-62979-z)
Supplement: Supplementary file 1 — Supplementary Tables. [file 41598_2024_62979_MOESM1_ESM.docx]

**Appendix A. Supplementary material**

**Table S1.** Sector consolidation method for China’s IO table from 2000 to 2020

| **Code** | **Sectors** | **Year 2000**  **(40 sectors)** | **Year 2002,** **2005, 2007, 2010 (42sectors)** | **Year 2012,** **2015, 2017, 2020**  **(42 sectors)** |  |
| --- | --- | --- | --- | --- | --- |
|  |  |  |  |  |  |
| S1 | Agriculture | Sector 1 | Sector 1 | Sector 1 |  |
| S2 | Coal mining | Sector 2 | Sector 2 | Sector 2 |  |
| S3 | Petroleum and natural gas | Sector 3 | Sector 3 | Sector 3 |  |
| S4 | Metal | Sector 4 | Sector 4 | Sector 4 |  |
| S5 | Non-metal | Sector 5 | Sector 5 | Sector 5 |  |
| S6 | Food and tobacco | Sector 6 | Sector 6 | Sector 6 |  |
| S7 | Textile | Sector 7-8 | Sector 7-8 | Sector 7-8 |  |
| S8 | Timber processing | Sector 9-10 | Sector 9-10 | Sector 9-10 |  |
| S9 | Petroleum processing | Sector 11 | Sector 11 | Sector 11 |  |
| S10 | Chemical industry | Sector 12 | Sector 12 | Sector 12 |  |
| S11 | Non-metal mineral products | Sector 13 | Sector 13 | Sector 13 |  |
| S12 | Non-ferrous metals | Sector 14-15 | Sector 14-15 | Sector 14-15 |  |
| S13 | Ordinary machinery for special purpose | Sector 16 | Sector 16 | Sector 16-17 |  |
| S14 | Transportation | Sector 17 | Sector 17 | Sector 18 |  |
| S15 | Electric equipment | Sector 18-19 | Sector 18-20 | Sector 19-21 |  |
| S16 | Other manufacturing activities | Sector 21-23 | Sector 21-22 | Sector 22-24 |  |
| S17 | Electric power and heat production and supply | Sector 24-25 | Sector 23-25 | Sector 25-27 |  |
| S18 | Construction | Sector27 | Sector 26 | Sector 28 |  |
| S19 | Transport and postal service | Sector 28-29 | Sector 27-28 | Sector 30 |  |
| S20 | Other service and activities | Sector 30-40 | Sector 29-42 | Sector 29、31-42 |  |

Note: The original sectoral information is obtained from the National Bureau of Statistics of China. The year 2000 originally have 40 sectors, the year 2002 originally have 128 sectors, the year 2007 originally have 135 sectors, the year 2012 originally has 139 sectors, the year 2017 originally have 149 sectors, and the year 2020 originally have 153 sectors. The year 2005, 2010 and 2015 originally have 42 sectors

**Table S2.** Changes in embodied CH_4_ emissions by final demand category during 2000–2020 (Tg)

| Years | Consumption | | | Investment | | Export | Total |
| --- | --- | --- | --- | --- | --- | --- | --- |
|  | Rural Consumption | Urban Consumption | Government Consumption | Capital Formation | Stock Increase | Export |  |
| 2000 | 12.9 | 12.5 | 3.4 | 6.3 | 0.0 | 5.9 | 41.1 |
| 2002 | 8.2 | 13.5 | 3.7 | 8.2 | 1.1 | 6.7 | 41.4 |
| 2005 | 7.0 | 13.9 | 4.3 | 11.4 | -0.1 | 11.9 | 48.4 |
| 2007 | 6.7 | 14.9 | 4.1 | 11.1 | 1.4 | 12.7 | 50.9 |
| 2010 | 6.2 | 15.5 | 4.1 | 15.2 | 1.4 | 11.2 | 53.6 |
| 2012 | 6.3 | 16.8 | 4.1 | 16.0 | 2.2 | 10.9 | 56.2 |
| 2015 | 6.2 | 17.3 | 4.4 | 17.8 | 1.4 | 10.1 | 57.1 |
| 2017 | 6.0 | 18.9 | 4.6 | 16.5 | 0.7 | 8.9 | 55.6 |
| 2020 | 6.2 | 19.5 | 5.5 | 18.3 | 0.9 | 9.6 | 60.0 |

**Table S3a.** Distributions of sectoral embodied CH_4_ emissions in the production layer in 2000

| Sector | Embodied CH_4_ emissions | Distribution of embodied CH_4_ emissions (%) | | | | | Total |
| --- | --- | --- | --- | --- | --- | --- | --- |
|  | (Tg) | PL^0^ | PL^1^ | PL^2^ | PL^3^ | PL^4→∞^ |  |
| S1 | 15.1 | 77.1% | 12.5% | 5.3% | 2.5% | 2.7% | 100% |
| S2 | 0.6 | 95.0% | 2.6% | 1.1% | 0.6% | 0.8% | 100% |
| S3 | 0.2 | 75.0% | 3.8% | 11.7% | 4.1% | 5.4% | 100% |
| S4 | 0.0 | 0.0% | 17.7% | 36.8% | 19.6% | 25.8% | 100% |
| S5 | 0.0 | 0.0% | 31.8% | 29.2% | 16.3% | 22.7% | 100% |
| S6 | 5.7 | 0.2% | 64.9% | 19.2% | 7.8% | 8.0% | 100% |
| S7 | 2.2 | 0.0% | 32.9% | 24.9% | 16.9% | 25.3% | 100% |
| S8 | 0.5 | 0.4% | 32.3% | 25.4% | 17.1% | 24.9% | 100% |
| S9 | 0.1 | 0.1% | 67.2% | 11.9% | 10.4% | 10.4% | 100% |
| S10 | 1.0 | 0.5% | 25.7% | 32.1% | 18.5% | 23.1% | 100% |
| S11 | 0.3 | 0.0% | 41.4% | 26.7% | 13.8% | 18.1% | 100% |
| S12 | 0.4 | 0.7% | 20.5% | 33.2% | 19.7% | 25.9% | 100% |
| S13 | 0.7 | 0.0% | 16.7% | 24.3% | 22.8% | 36.3% | 100% |
| S14 | 0.6 | 0.0% | 8.8% | 20.0% | 22.7% | 48.5% | 100% |
| S15 | 1.4 | 0.0% | 7.0% | 20.8% | 25.1% | 47.1% | 100% |
| S16 | 0.1 | 0.0% | 39.6% | 20.4% | 15.5% | 24.5% | 100% |
| S17 | 0.6 | 0.2% | 74.3% | 14.4% | 4.7% | 6.4% | 100% |
| S18 | 3.7 | 0.5% | 11.1% | 33.0% | 22.9% | 32.5% | 100% |
| S19 | 0.2 | 7.0% | 17.9% | 33.2% | 15.4% | 26.6% | 100% |
| S20 | 7.6 | 40.4% | 19.1% | 17.3% | 9.7% | 13.5% | 100% |
| **Total** | **41.1** |  |  |  |  |  |  |

Table S3b. Distributions of sectoral embodied CH_4_ emissions in the production layer in 2010

| Sector | Embodied CH_4_ emissions | Distribution of embodied CH_4_ emissions (%) | | | | | Total |
| --- | --- | --- | --- | --- | --- | --- | --- |
|  | (Tg) | PL^0^ | PL^1^ | PL^2^ | PL^3^ | PL^4→∞^ |  |
| S1 | 7.5 | 77.0% | 10.4% | 5.7% | 3.0% | 3.9% | 100% |
| S2 | 0.5 | 80.8% | 13.2% | 3.0% | 1.2% | 1.8% | 100% |
| S3 | 0.1 | 78.7% | 3.7% | 6.9% | 4.2% | 6.5% | 100% |
| S4 | 0.0 | 0.0% | 6.9% | 37.1% | 23.5% | 32.4% | 100% |
| S5 | 0.0 | 0.0% | 5.6% | 33.9% | 23.9% | 36.6% | 100% |
| S6 | 7.7 | 0.2% | 55.9% | 21.1% | 10.3% | 12.6% | 100% |
| S7 | 3.4 | 0.0% | 29.6% | 23.4% | 16.9% | 30.1% | 100% |
| S8 | 1.0 | 0.4% | 28.4% | 22.7% | 17.3% | 31.2% | 100% |
| S9 | 0.4 | 0.1% | 69.5% | 13.1% | 7.2% | 10.1% | 100% |
| S10 | 1.6 | 0.9% | 23.2% | 27.0% | 18.8% | 30.1% | 100% |
| S11 | 0.4 | 0.0% | 38.7% | 25.2% | 14.7% | 21.4% | 100% |
| S12 | 1.0 | 1.0% | 23.5% | 25.0% | 18.9% | 31.5% | 100% |
| S13 | 2.2 | 0.0% | 7.3% | 21.9% | 22.5% | 48.3% | 100% |
| S14 | 2.0 | 0.0% | 4.8% | 17.2% | 21.3% | 56.6% | 100% |
| S15 | 3.7 | 0.0% | 4.9% | 19.4% | 22.6% | 53.1% | 100% |
| S16 | 0.5 | 0.0% | 36.0% | 21.4% | 15.6% | 27.0% | 100% |
| S17 | 1.3 | 0.3% | 50.4% | 25.8% | 11.4% | 12.2% | 100% |
| S18 | 8.6 | 0.6% | 5.4% | 29.3% | 24.3% | 40.3% | 100% |
| S19 | 0.7 | 4.1% | 18.3% | 36.1% | 15.7% | 25.8% | 100% |
| S20 | 10.9 | 34.8% | 12.6% | 18.1% | 13.0% | 21.5% | 100% |
| **Total** | **53.6** |  |  |  |  |  |  |

Table S3c. Distributions of sectoral embodied CH_4_ emissions in the production layer in 2020

| Sector | Embodied CH_4_ emissions | Distribution of embodied CH_4_ emissions (%) | | | | | Total |
| --- | --- | --- | --- | --- | --- | --- | --- |
|  | (Tg) | PL^0^ | PL^1^ | PL^2^ | PL^3^ | PL^4→∞^ |  |
| S1 | 9.2 | 78.2% | 10.7% | 5.2% | 2.7% | 3.2% | 100% |
| S2 | -0.1 | 83.8% | 12.2% | 2.4% | 0.8% | 0.8% | 100% |
| S3 | 0.1 | 94.8% | 0.4% | 1.7% | 1.4% | 1.6% | 100% |
| S4 | 0.0 | 0.0% | 14.6% | 42.2% | 20.9% | 22.2% | 100% |
| S5 | 0.0 | 0.0% | 22.6% | 38.1% | 18.3% | 21.0% | 100% |
| S6 | 6.7 | 0.2% | 58.2% | 20.9% | 9.7% | 11.0% | 100% |
| S7 | 2.1 | 0.0% | 30.0% | 23.8% | 17.6% | 28.6% | 100% |
| S8 | 1.2 | 0.3% | 30.1% | 23.7% | 18.0% | 27.9% | 100% |
| S9 | 0.8 | 0.1% | 79.3% | 11.4% | 4.3% | 4.9% | 100% |
| S10 | 1.7 | 1.4% | 30.3% | 29.6% | 17.5% | 21.2% | 100% |
| S11 | 0.4 | 0.0% | 43.2% | 27.0% | 14.2% | 15.6% | 100% |
| S12 | 1.0 | 1.2% | 23.2% | 29.9% | 20.1% | 25.6% | 100% |
| S13 | 1.8 | 0.0% | 5.8% | 22.1% | 25.0% | 47.1% | 100% |
| S14 | 1.5 | 0.0% | 4.5% | 18.7% | 24.0% | 52.8% | 100% |
| S15 | 2.7 | 0.0% | 3.8% | 20.4% | 24.7% | 51.1% | 100% |
| S16 | 0.1 | 0.0% | 19.3% | 26.7% | 21.2% | 32.8% | 100% |
| S17 | 2.6 | 0.5% | 57.0% | 25.1% | 9.5% | 7.8% | 100% |
| S18 | 13.2 | 0.4% | 6.8% | 32.3% | 25.3% | 35.2% | 100% |
| S19 | 1.2 | 4.0% | 5.8% | 42.4% | 20.3% | 27.4% | 100% |
| S20 | 13.8 | 26.9% | 13.1% | 19.6% | 15.6% | 24.9% | 100% |
| **Total** | **60.0** |  |  |  |  |  |  |

**Table S4.** Selected studies on supply chain analysis on China’s embodied CH_4_ emissions

| Publication | Sample period | Top ranked path for CH_4_ | Top 3 sectors in final demand | Main result |
| --- | --- | --- | --- | --- |
| Zhang et al. (2018b) | 2012 | Farming, Forestry, Animal Production and Fishery→Urban Consumption | 1. Construction 2. Farming, Forestry, Animal Production and Fishery 3. Manufacture of food and tobacco | By examining both production and consumption-based non-CO_2_ GHG emissions. A deeper understanding of trades in non-CO_2_ GHG emissions by considering the abatement potentials within supply chains can be gained. |
| Zhang et al. (2021) | 2012 | Farming, Forestry, Animal Production and Fishery→Urban Consumption | 1. Farming, Forestry, Animal Production and Fishery 2. Manufacture of food and tobacco 3. Wholesale, Retail  Trade, Hotels, Catering Service. | Controlling important supply chain paths and critical transmission sectors can reduce emissions of CH_4_ while saving water resources. |
| Long et al. (2022) | 2017 | Mining and washing of coal →Manufacture of cement, Lime and plaster→Construction of buildings→Gross fixed capital formation, | 1 Manufacture of machinery for mining, metallurgy and construction 2. Manufacture of lifting and handling equipment 3. Air passenger transport | The energy-water-GHGs nexus is a critical link in China's resource management and emission control in supply chain. |
